# Activity-Dependent Phosphorylation by CaMKIIδ Alters the Ca^2+^ Affinity of the Multi-C_2_-Domain Protein Otoferlin

**DOI:** 10.3389/fnsyn.2017.00013

**Published:** 2017-10-04

**Authors:** Sandra Meese, Andreia P. Cepeda, Felix Gahlen, Christopher M. Adams, Ralf Ficner, Anthony J. Ricci, Stefan Heller, Ellen Reisinger, Meike Herget

**Affiliations:** ^1^Department of Molecular Structural Biology, Institute for Microbiology and Genetics, and Collaborative Research Center 889, University of Göttingen, Göttingen, Germany; ^2^Göttingen Graduate School for Neurosciences, Biophysics, and Molecular Biosciences, Göttingen, Germany; ^3^Molecular Biology of Cochlear Neurotransmission Group, Department of Otorhinolaryngology, University Medical Center Göttingen, and Collaborative Research Center 889, University of Göttingen, Göttingen, Germany; ^4^Department of Otorhinolaryngology, Head and Neck Surgery, Ruhr-University Bochum, Bochum, Germany; ^5^Stanford University Mass Spectrometry, Stanford University, Stanford, CA, United States; ^6^Department of Otolaryngology, Head and Neck Surgery, Stanford School of Medicine, Stanford, CA, United States

**Keywords:** C_2_ domains, hair cell, synaptic transmission, Ca^2+^ affinity, phosphorylation, CaMKII

## Abstract

Otoferlin is essential for fast Ca^2+^-triggered transmitter release from auditory inner hair cells (IHCs), playing key roles in synaptic vesicle release, replenishment and retrieval. Dysfunction of otoferlin results in profound prelingual deafness. Despite its crucial role in cochlear synaptic processes, mechanisms regulating otoferlin activity have not been studied to date. Here, we identified Ca^2+^/calmodulin-dependent serine/threonine kinase II delta (CaMKIIδ) as an otoferlin binding partner by pull-downs from chicken utricles and reassured interaction by a co-immunoprecipitation with heterologously expressed proteins in HEK cells. We confirmed the expression of CaMKIIδ in rodent IHCs by immunohistochemistry and real-time PCR. A proximity ligation assay indicates close proximity of the two proteins in rat IHCs, suggesting that otoferlin and CaMKIIδ also interact in mammalian IHCs. *In vitro* phosphorylation of otoferlin by CaMKIIδ revealed ten phosphorylation sites, five of which are located within C_2_-domains. Exchange of serines/threonines at phosphorylated sites into phosphomimetic aspartates reduces the Ca^2+^ affinity of the recombinant C_2_F domain 10-fold, and increases the Ca^2+^ affinity of the C_2_C domain. Concordantly, we show that phosphorylation of otoferlin and/or its interaction partners are enhanced upon hair cell depolarization and blocked by pharmacological CaMKII inhibition. We therefore propose that otoferlin activity is regulated by CaMKIIδ in IHCs.

## Introduction

Otoferlin is a 230 kDa, tail-anchored membrane protein, containing at least six C_2_ domains implicated in Ca^2+^, phospholipid, and protein binding (Yasunaga et al., 1999; Johnson and Chapman, 2010; Pangršič et al., 2012). Dysfunction of otoferlin underlies DFNB9, a recessive and non-syndromic form of prelingual deafness in humans characterized by impaired synaptic transmission from IHCs (Yasunaga et al., 1999). Unique to IHC ribbon synapses, otoferlin is hypothesized to operate as a Ca^2+^-sensor in synaptic vesicle fusion (Roux et al., 2006), and it was shown to be involved in vesicle replenishment, vesicle reformation from bulk endosomes, active zone clearance, and clathrin-mediated endocytosis (Pangršič et al., 2010; Duncker et al., 2013; Jung et al., 2015; Strenzke et al., 2016). To date, several protein interaction partners of otoferlin have been reported including myosin VI, Rab8b, SNARE proteins, Cav1.3 Ca^2+^ channel, Ergic2 and AP-2 (Roux et al., 2006; Heidrych et al., 2008, 2009; Ramakrishnan et al., 2009; Roux et al., 2009; Zak et al., 2012; Duncker et al., 2013; Jung et al., 2015). However, the physiological effects of many of these interactions remain only partially understood. In this study, we aimed to identify new otoferlin interaction partners and to address a potential role of these interactions in IHC synaptic function.

Neurotransmitter release from IHCs is extraordinary in several respects. Firstly, it is precisely coupled to the cycle of auditory sine waves generating graded receptor potentials in IHCs up to 3 kHz in rodents (Palmer and Russell, 1986). Secondly, release is largely indefatigable with a sustained vesicle fusion rate of up to 2300 vesicles per second per active zone (Strenzke et al., 2016). Thirdly, exocytosis elicits large EPSCs to reliably trigger postsynaptic spikes (Glowatzki and Fuchs, 2002; Rutherford et al., 2012), and fourthly, exocytosis from IHCs does not require neuronal SNARE proteins (Nouvian et al., 2011). Remarkably, the 10–20 ribbon synapses in each IHC respond differently to the same graded depolarization, a process required to encode different sound intensities, the molecular mechanisms of which are only beginning to be understood (Merchan-Perez and Liberman, 1996; Taberner and Liberman, 2005; Frank et al., 2009; Meyer et al., 2009; Hickman et al., 2015; Ohn et al., 2016; Reijntjes and Pyott, 2016).

Presynaptic activity was reported to be regulated in many synapses of the central nervous system and in sensory systems, e.g., by phosphorylation of presynaptic proteins, thereby leading to adaptation (or facilitation) to constant stimuli. The auditory system does not grossly adapt, at least not to mild or moderate sound stimuli. In contrast, exposure to noise can cause both temporary and permanent threshold shifts, depending on stimulus levels and duration (Kujawa and Liberman, 2009). While a number of mechanisms have been suggested to underlie a temporary threshold shift, adaptation of the presynaptic machinery has not been studied to date.

Here, we studied the interaction of the presynaptic IHC protein otoferlin with CaMKIIδ, the induction of otoferlin phosphorylation and the effects of phosphorylation on Ca^2+^ binding. Our data indicate that the functions of otoferlin in exocytosis, vesicle replenishment and endocytosis might be regulated during strong IHC stimulation.

## Materials and Methods

### Animal Welfare

Animal handling complied with national animal care guidelines. For rats and chicken, handling was approved by the Administrative Panel on Laboratory Animal Care (APLAC) of Stanford University and accredited by the Association for the Assessment and Accreditation of Laboratory Animal Care (#A3213-01). For mice, handling was approved by the University of Göttingen Board for animal welfare and the animal welfare office of the state of Lower Saxony, Germany.

### Co-immunoprecipitation of Otoferlin from Chicken Utricle Hair Cells

Utricles were dissected from embryonic day 18 (E18) old chicken. Otoconial membranes and spiral ganglion nerve fibers were removed and utricles were collected into chilled lysis buffer (50 mM Tris-HCl, 140 mM NaCl, 5% Glycerol, 250 mM sucrose, protease inhibitors (Roche, EDTA-free), pH 7.4). The tissue was homogenized by triturating six times through a 26 gauge needle, followed by centrifugation for 5 min at 600 × g at 4°C (Eppendorf tabletop Centrifuge 5417C). The supernatant was subsequently centrifuged at 100000 × *g* for 30 min at 4°C to pellet membranes (Beckmann, TL-100 Ultracentrifuge). The pellet was resuspended in solubilization buffer (20 mM Tris-HCl, 10% glycerol, 140 mM NaCl, 1% octyl-β-D-glucopyranoside, protease inhibitors, pH 7.4) for 1 h on ice. After solubilization, samples were centrifuged for 20 min at 55000 × *g* at 4°C (Beckmann, TL-100 Ultracentrifuge). Supernatants were incubated with 25 μL Dynabeads (Life Technologies, Dynabeads M-270 Epoxy) conditioned with either 5 μg chicken HCS-1 antibody (mouse, monoclonal; Goodyear et al., 2010) or 5 μg control antibody, TLA (tip-link antigen, mouse monoclonal; Goodyear and Richardson, 2003), according to the manufacturer’s protocol. Immunoprecipitation of otoferlin was performed for 2 h at 4°C. Beads were washed three times for 15 min at 4°C with solubilization buffer containing 0.1% octyl-β-D-glucopyranoside. Beads were then boiled for 5 min at 95°C in Laemmli sample buffer (BioRad), loaded onto a 4–20% SDS PAGE (BioRad) and proteins were allowed to run 1 cm into the separation gel and visualized with Coomassie brilliant blue staining (BioRad).

### Mass Spectrometric Analysis of Immunoprecipitated Otoferlin and Interacting Proteins

Gel bands were excised and digested in-gel using trypsin (Promega) as previously described (Shevchenko et al., 2006). Dried peptides were reconstituted in 0.1% formic acid, 2% acetonitrile and 97.9% water. Peptides were loaded onto a self-packed C18 reverse phase column with an ID of 100 μM and 15 cm in length. Over the course of all LC-MS/MS experiments, two LCs were used: a nanoAcquity UPLC (Waters) and a nano2D LC (Eksigent, AB Sciex), with flow rates of 300 nL/min and mobile phase A consisting of 0.585% (vol/vol) acetic acid in water and mobile phase B of 0.585% (vol/vol) acetic acid and 2% (vol/vol) water in acetonitrile. The mass spectrometer (LTQ Orbitrap Velos, Thermo Fisher) utilized data-dependent acquisition in which the top 12 most intense precursor ions were selected for fragmentation. The raw data were converted to mzXML format and searched against the UniProt *Gallus gallus* database using *Sequest* on a sorcerer platform (Sage-N). Search parameters included tryptic specificity, allowing for a maximum of two miscleavages with static modification of propionamide (cysteine) and variable modifications of oxidation (methionine), phosphorylation (serine, threonine, tyrosine) and alkylation (lysine). The precursor mass tolerance was 20 ppm, and the data was further filtered using the *Scaffold* software (Proteome Software). In the case of identified interacting partners we stringently required at least 4 unique peptides with a 95% peptide threshold and 99.9% protein probability threshold, thereby effectively filtering out all non-specific contaminants.

### Real-time PCR Experiments

PCRs on a few IHCs were performed essentially as described (Kerr et al., 2008; Reisinger et al., 2011). Organs of Corti (OCs) from P14 mice were dissected in HEPES-Hanks solution (5.36 mM KCl, 141.7 mM NaCl, 1 mM MgCl_2_, 0.5 mM MgSO_4_, 10 mM Na-HEPES, 6.84 mM L-glutamine, 5.55 mM D-glucose, pH 7.2) and perfused with modified Ringer’s solution thereafter (113 mM NaCl, 35 mM TEA-Cl, 2.8 mM KCl, 2 mM CaCl_2_, 1 mM MgCl2, 10 mM Na-HEPES, 1 mM CsCl, 11.1 mM D-glucose; pH adjusted to 7.2, osmolarity approximately 300 mOsm). Outer hair cells and supporting cells were removed with glass capillaries in a patch-clamp setup. Once inner phalangeal cells were removed, 3–5 IHCs were collected in one patch clamp glass capillary filled with 8 μL KCl solution (140 mM KCl, 5 mM K-HEPES, 5 mM EGTA, 3 mM MgCl_2_, pH 7.3). Before and after collecting cells, bath controls were taken by lowering the patch pipette close to the tissue and removing overpressure for 5–10 s. The content of each capillary was expelled into a reaction tube containing buffer for reverse transcription [2.5 μL first strand buffer, 0.6 μL Oligo(dT)_20_ primer (50 μM), 0.5 μL Random hexamers (50 ng/μL), 0.7 μL dNTP mix (10 mM each), 1.4 μL DTT (0.1 M) and 0.8 μL Ribonuclease Inhibitor (40 U/μL)]. The reaction was started by adding 0.5 μL SuperScript^®^ IV Reverse Transcriptase (100 units; Thermo Fisher Scientific) and incubated for 10 min at room temperature (RT), followed by 20 min at 37°C and 2 h at 42°C. The resulting cDNA was precipitated over night at -20°C in 70% EtOH and 1 μL glycogen (Ambion), washed with 70% EtOH, dried and resuspended in 25 μL H_2_O. The cDNA solution from each sample was split into six PCR reactions. cDNA quality was assessed with TaqMan assays for bassoon (Mm00464451_m1; Applied Biosystems) and TATA-binding protein (Mm00446973_m1; Applied Biosystems). To test for CaMKII isoforms in a SYBR green assay, we designed intron-overspanning amplicons targeting all splice variants of the four CaMKII genes (in *Mus musculus*) described as reference sequences in NCBI databases. Amplification efficiency of the assays was determined with standard curve assays, resulting in 95.7–96.9% efficiencies for all transcripts. We used the following oligonucleotides: CaMKIIα: 5′-GAAGATGTGCGACCCTGGAA-3′ and 5′-TGATGCGGATATAGGCGATG-3′ (400 nM each), CaMKIIβ: 5′-ACAAACAGCACCAAAAACAGCT-3′ and 5′-GAGCTGCTCTGTGGTCTTGA-3′ (300 nM each), CaMKIIγ: 5′-TTACGCAAATTCAACGCCCG-3′ and 5′-GACACCGCCATCTGACTTCT-3′ (400 nM each), CaMKIIδ: 5′-CGTCTCTTGAAGCACCCCAA-3′ and 5′-AAACAGTTCGCCACCAGTCA-3′ (300 nM each). Mouse brain cDNA was used as positive control. Amplification with 2x TaqMan universal PCR Mastermix (Applied Biosystems) or 2x Power SYBR green Mastermix (Applied Biosystems) was conducted in an Applied Biosystems 7500 Real Time PCR system using default PCR parameters. Dissociation curve assays revealed the melting temperatures to control for amplicon specificity. In addition, we assayed the size of all amplicons with gel electrophoresis on 2% agarose gels with EtBr staining. We analyzed only those IHC samples for CaMKII expression where both bassoon and TATA-binding protein transcripts were detected.

### Immunohistochemistry

**FIGURE 1:**
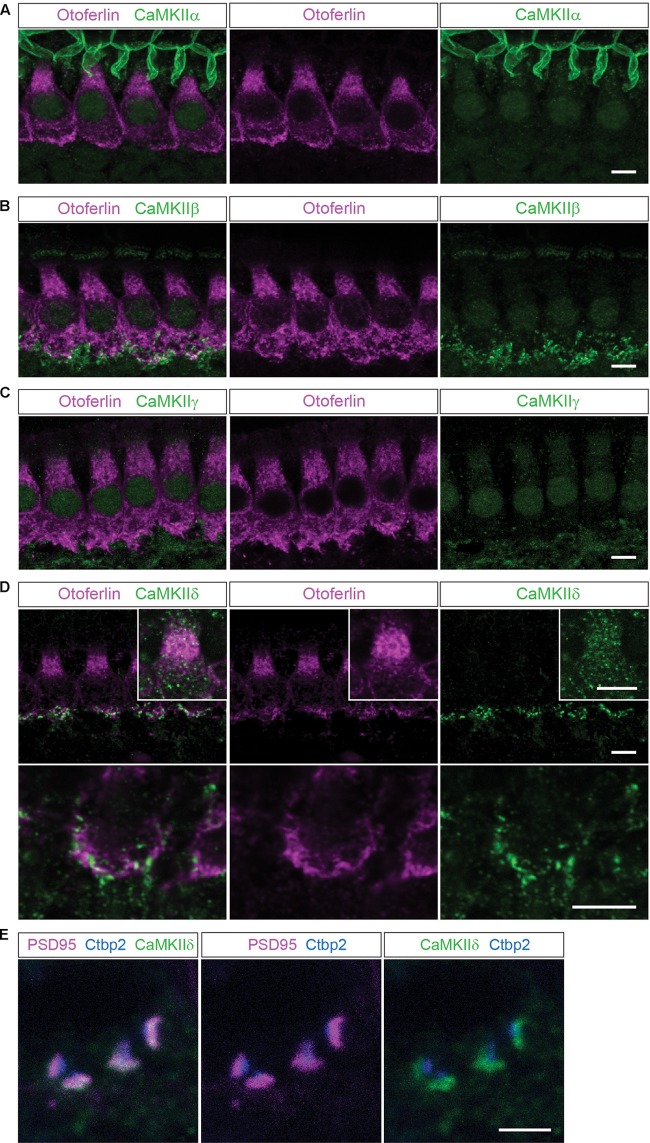
Localization of the different CaMKIIs in the organ of Corti. **(A)** Single optical sections acquired by confocal microscopy display immunolabelling of CaMKIIα (green) in P14 mouse IHCs, co-labeled for otoferlin (magenta). Here, no definite expression in IHCs could be detected. **(B–E)**, CaMKIIβ, γ and δ are present in structures outside the IHCs, possibly in efferent and/or afferent synaptic boutons. **(D)**, CaMKIIδ can additionally be detected in the cytoplasm of IHCs, visualized in insets with enhanced CaMKIIδ fluorescence. Scale bars in **(A–D)**, 5 μm. **(E)**, co-labeling against the ribbon marker Ctbp2 and the postsynaptic protein PSD95 indicates a localization of CaMKIIδ at the postsynaptic site of afferent synaptic boutons; scale bar 2 μm.

Sprague Dawley rats at P9 to P11 or P14 C57Bl6J mice of either gender were decapitated and cochleae were dissected in chilled Hank’s balanced salt solution (HBSS, HyClone). OCs were fixed in chilled 3% paraformaldehyde for 25 min, permeabilized with 0.5% Triton X-100 in PBS for 30 min and incubated in blocking solution (1% BSA and 0.1% Triton X-100 in PBS) for 1 h at RT. Antibodies to otoferlin (mouse, 1:400; Abcam, ab53233), myosin VI (rabbit, 1:400; Sigma, M5187), parvalbumin (1:2000, raised against bullfrog parvalbumin-3, in mice it recognizes oncomodulin and parvalbumin; Heller et al., 2002), CaMKIIα (rabbit, 1:300; Sigma–Aldrich, C6974), CaMKIIβ (rabbit, 1:300; Abcam, ab34703), CaMKIIγ (rabbit, 1:300; Acris, AP13886PU-N), CaMKIIδ (rabbit, 1:300; Genetex, GTX111401), pan-CaMKII (rabbit, 1:300; Abcam, ab52476), PSD95 (mouse, 1:500, Abcam, ab2723), Ctbp2 (goat, 1:200, Santa Cruz, sc-5966) and phosphoserine (rabbit, 1:300; Abcam, 9332), were diluted in blocking solution and incubated on OCs overnight at 4°C. Alexa fluor 488, 546, 568, and 633-conjugated secondary antibodies (1:200-1:600; Thermo Fisher Scientific) were used. The tissue was mounted with Aqua Poly/Mount (Polysciences) and for **Figures 3**, **4**, and **10** imaged using an AxioImager/LSM 5 Exciter confocal microscope (Zeiss). Images in **Figure 1** were acquired using a Leica SP5 confocal microscope with 63x glycerol objective (NA 1.456).

### Proximity Ligation Assay

P9–P11 rat OCs were dissected in chilled external solution (10 mM HEPES, 2 mM MgCl_2_, 2 mM CaCl_2_, 2 mM KCl, 145 mM NaCl, 6 mM D-glucose, 2 mM ascorbate, 2 mM pyruvate, 2 mM creatine, pH 7.4) and fixed in chilled 3% paraformaldehyde for 25 min. For hair cell stimulation, acutely dissected OCs were transferred into prewarmed high K^+^ external solution (KCl increased to 40 mM, NaCl reduced to 35 mM) and incubated for 15 min at 37°C and 5% CO_2_ before fixation. To pharmacologically inhibit CaMKII, OCs were incubated for 10 min in prewarmed external solution supplemented with 50 μM of selective inhibitor KN-93 (Cayman Chemical) followed by stimulation with high K^+^ external solution + KN-93 and fixation. A proximity ligation assay (PLA) (Duolink, Sigma) was performed with mouse otoferlin (1:500; Abcam, ab53233) antibody in combination with rabbit myosin VI (1:500; Sigma, M5187), rabbit parvalbumin (1:2000; Heller et al., 2002), rabbit CaMKIIδ (1:300; Genetex, GTX111401), rabbit phosphoserine (1:300; Abcam, 9332), and rabbit pan-CaMKII (1:300; Abcam, ab52476). The manufacturer’s protocol was applied with the following modifications: Fixed OCs were semipermeabilized in 0.5% Triton X-100 at RT for 30 min and subsequently blocked with Blocking buffer (Duolink, Sigma) for 2 h at RT. Primary antibodies were diluted in antibody diluent (Duolink, Sigma) and incubated overnight at 4°C. The tissue was then washed four times with gentle shaking in 2 mL Buffer A (Duolink, Sigma). PLA probes (anti mouse MINUS and anti-rabbit PLUS; Duolink, Sigma) were diluted 1:6 in 30 μL antibody diluent and incubated for 1 h at 37°C and 5% CO_2_. Tissue was washed four times with 2 mL Buffer A and gentle shaking, followed by incubation with 30 μL ligation mix for 30 min at 37°C and 5% CO_2_. After three washes for 10 min in 2 mL Buffer A and gentle shaking, 30 μL DNA amplification mix was added and incubated for 100 min at 37°C and 5% CO_2_. After amplification, the tissue was washed twice for 10 min in 1x Buffer B (Duolink, Sigma) followed by two times washing in 2 mL 0.01x Buffer B. To visualize hair cells, tissue was subsequently stained with Alexa Phalloidin 488 (Invitrogen) (1:200 in 0.01x Buffer B) for 15 min at RT, followed by two 10 min washing steps in 2 mL 0.01x Buffer B. For counterstainings of synaptic ribbons, anti-Ctbp2 antibody (goat, 1:200, Santa Cruz, sc-5966) was incubated together with primary antibodies to otoferlin and phosphoserine, and secondary anti-goat antibodies were co-incubated with PLA probes. For imaging, tissue was mounted with DAPI containing Duolink In Situ Mounting media. Images were taken with a LSM700 confocal microscope (Zeiss) with Zen software (Zeiss).

### Quantification of PLA Signals and Statistical Analysis

To quantify PLA signal intensities in confocal images, IHCs were outlined manually using Image J software and pixel intensities of the fluorescent PLA signals were determined. For each experimental condition three independent experiments were performed and a total of 30 IHCs were analyzed. The mean pixel intensity per IHC was calculated for each condition and compared. A two-tailed *t*-test was applied to assess a statistical significance of the changes in PLA signal pixel intensities amid the different experimental conditions.

### *In Vitro* Pull-Down Assays

Two mouse otoferlin fragments (C_2_ABC: aa 1–632, 70 kDa and C_2_DEF: aa 933–1920, 114 kDa) were PCR amplified from cDNA encoding full-length mouse otoferlin (NM_001100395) with a C-terminal HA-tag using the following primer pairs: C_2_ABC-HA (5′-GAATTCACCATGGCCCTGATTGTTCACCT-3′, 5′-GCGGCCGCCTAAGCGTAATCTGGAACATCGTATGGGTACATGGTTCCTCCTGTGCAGCTCTCCGAGACAG-3′); C_2_DEF (5′-GAATTCACCATGAGCAAGCAG-CGAAAGGACTTC-3′, 5′-GCGGCCGCCTAAGCGTAATCTGGAACATCGTATG-GGTACATGGTTCCTCCGCGAGCCAGGCCCACAGGG-3′). Fragments were subcloned into the pCl mammalian expression vector (Promega) via NotI and EcoRI. Full-length CaMKIIδ (NM_001025438.1) was amplified from mouse postnatal day 6 (P6) cochlea cDNA (5′-GAATTCACCATGGCTTCGACCACCACCTG-3′, 5′-GGTACCCCGATGTTTTG-CCACAAAGAGG-3′) and subcloned as N-terminal fusion construct into the pmCherry-N1 mammalian expression vector (Clontech) via EcoRI and KpnI. For co-immunoprecipitation (co-IP) of otoferlin, HEK293 cells were grown in a 10 cm culture dish to 80% confluency and transiently co-transfected with HA-tagged C_2_ABC, HA-tagged C_2_DEF and mcherry-tagged CaMKIIδ. 40 h post-transfection, cells were collected in TBS buffer (25 mM Tris-HCl, 150 mM NaCl, protease inhibitors (Roche, complete, EDTA free), pH 7.4), lysed by triturating 5 times through a 26^1/2^ gauge needle, and centrifuged for 5 min at 4°C at 500 × *g* to remove debris. For co-IP of CaMKIIδ-mcherry with otoferlin C_2_ABC-HA and/or C_2_DEF-HA, the lysate was mixed with anti-HA agarose slurry (35 μg anti-HA antibody, Pierce HA Tag IP/Co-IP Kit, Thermo Scientific) and incubated with gentle end-over-end mixing for 2 h at 4°C. Agarose was washed four times with TBS-T buffer (25 mM Tris-HCl, 150 mM NaCl, 0.05% Tween, pH 7.4) before boiling for 5 min at 95°C in Laemmli sample buffer and applied on a 4–20% SDS PAGE gel (BioRad). Protein complexes were analyzed by immunoblots using a Trans Blot semi-dry transfer cell (BioRad) and polyclonal anti-HA antibodies (1:1000; Rockland, 600-401-384) and monoclonal anti-RFP antibodies (1:20000; Rockland, 200-301-379). Secondary anti-rabbit Dylight680 and anti-mouse Dylight800 antibodies (1:10000; Rockland, 611-144-003 and 610-145-003) were incubated for 1 h at RT, and after washing the blots for three times with TBS-T buffer, fluorescent signals were detected using a Li-Cor Odyssey system.

### Recombinant Expression of Otoferlin Fragments

Two soluble mouse otoferlin domains comprising either the first three C_2_ domains (C_2_ABC), or the last three C_2_ domains (C_2_DEF) were heterologously expressed in *Escherichia coli (E. coli)* SoluBL21 (DE3). The C_2_ABC fragment (aa 1–616, 70 kDa; NP_001093865) was PCR amplified (5′-AGCGGCTCTTCAATG-ATGGCCCTGATTGTTCACCT-3′, 5′-AGCGGCTCTTCTCCC-CTCCGAGACAGGCGTGGC-3′) and subcloned with a C-terminal hexahistidine-tag into the bacterial expression vector pPSG-IBA33 (Iba Lifesciences) and expressed at 30°C. After induction with IPTG, the temperature was changed to 16°C and the culture was harvested 16–20 h post-induction.

The cells were collected in lysis buffer (70 mM HEPES pH 7.4, 300 mM NaCl, 10 mM imidazole) and lysed by fluidizing (microfluidizer S, Microfluidics, Westwood, MA, United States). After centrifugation at 20000 rpm (JA-20 fixed angle rotor, Beckmann Coulter) for 45 min at 4°C, the supernatant was loaded onto a Ni-NTA-column (GE Healthcare). After washing, the recombinant proteins were eluted by gradient elution with imidazole containing buffer (70 mM HEPES, 300 mM NaCl, 500 mM imidazole, pH 7.4). For buffer exchange to 10 mM HEPES, 300 mM NaCl, pH 7.4, the proteins were further applied on a size exclusion chromatography column (HiPrep 16/60 Sephacryl S-200 HR, GE Healthcare). During all purification steps the temperature was kept at 4°C.

C_2_DEF (aa 908–1932, 118 kDa; NP_001093865) was PCR amplified (5′-GAGAGGATCCAAGCTGGAGCTCTACCTGTG-3′, 5′-GAGAGAATTCTAATCA-GGTTCATTGCGAGCCAG-3′) and subcloned with a hexahistidine tag into the bacterial expression vector pET28a. Expression took place for 60 h at 16°C using an autoinduction system (Studier, 2005).

The C_2_DEF was purified from inclusion bodies by resuspending the cell pellet in lysis buffer. After centrifugation for 45 min at 4°C and 20000 rpm (JA-20 fixed angle rotor, Beckmann Coulter), the supernatant was discarded and the pellet was washed three times with PBS buffer (4 mM KH_2_PO_4_, 16 mM Na_2_HPO_4_, 115 mM NaCl, pH 7.4), containing 1% Triton in the first washing step. The pellets were frozen overnight at -20°C and subsequently dissolved in Urea buffer (70 mM HEPES, 300 mM NaCl, 10 mM imidazole, 8 M urea, pH 7.4). After centrifugation, the supernatant was loaded onto a Ni-NTA-column (GE Healthcare) and recombinant proteins were eluted with an imidazole gradient.

### *In Vitro* Phosphorylation Assay

For *in vitro* phosphorylation of recombinant otoferlin, 21.5 pmol recombinant CaMKIIδ (Life Technologies) was incubated together with equimolar amounts of recombinant otoferlin C_2_ABC and C_2_DEF domains (1:1:1) in 30 μL assay buffer (10 mM HEPES, 10 mM MgCl_2_, 10 μg/mL calmodulin, 0.5 mM CaCl_2_, 5 mM DTT, 100 μM ATP, pH 7.5) for 5 min at 30°C. The reaction was inactivated by adding Laemmli-buffer and subsequent incubation at 95°C for 5 min. For control experiments, otoferlin C_2_ABC and C_2_DEF were incubated in assay buffer in the absence of CaMKIIδ.

### Mass-Spectrometric Analysis of Otoferlin Phosphorylation Sites

Gel bands corresponding to recombinant mouse otoferlin fragments, C_2_ABC (70 kDa) and C_2_DEF (118 kDa), were excised from the Coomassie gel after *in vitro* phosphorylation and prepared for LC-MS/MS analysis as described in above. During data acquisition the mass spectrometer was set to perform ion-trap MS/MS and high energy collision-induced dissociation (HCD) MS/MS on the same precursor masses to provide more complete fragmentation data and to increase the probability of correctly localizing the site of phosphorylation. All suggested phosphorylation sites were manually validated by interrogation of the fragment ion spectra, where neutral loss of phosphoric acid (H_3_PO_4_) was observed as well as site localization of the phosphor-group by corresponding *b* or *y* ions.

### Expression and Phosphomimetic Mutagenesis of C_2_C and C_2_F Domains

The protein fragments of otoferlin used for Ca^2+^ binding assays – C_2_C (aa 410–616 in pGEX-6P-3, NP_001263649) and C_2_F (aa 1695–1934 in pGEX-6P-3, NP_001263649.1) were expressed in *E. coli Rosetta 2 (DE3)* cells using the auto-induction system (Studier, 2005; Meese, 2015). The harvested cells were lysed in 75 mM HEPES pH 7.4, 300 mM NaCl using the microfluidizer S (Microfluidics, Westwood, MA, United States). The obtained supernatant after centrifugation was loaded onto 5 mL GST Trap columns (GE Healthcare). Using a glutathion containing buffer (75 mM HEPES pH 7.4, 300 mM NaCl, 25 mM reduced glutathion) the protein was eluted from the column and incubated with PreScission protease for 14 h at 4° C to cleave off the GST-tag. In the next step a size exclusion chromatography (S200 16/60, GE Healthcare) was performed (10 mM HEPES pH 7.4, 150 mM NaCl) followed by a GST trap column to separate the C_2_ domain from the GST-tag. The protein solution was incubated with Chelex (Biorad) for 1 h at 4°C, concentrated and stored at -80°C.

In order to mimic phosphorylation sites, we replaced phosphorylated serine/threonine residues with aspartate residues. The cDNA for the “C2F-pm” fragment (S1783D, S1814D, T1866D) was newly synthesized by GeneArt (Life Technologies). For the “C2C-pm” fragment a mutation (T434D) was inserted using a “QuikChange” site directed mutagenesis protocol (Agilent Technologies). The expression and purification procedure was the same as for the wild type proteins.

### Ca^2+^ Affinity Measurement by MicroScale Thermophoresis (MST)

For MicroScale Thermophoresis (MST) the NT.LabelFree instrument (NanoTemper Technologies GmbH, Munich, Germany) was used. All solutions were treated with Chelex (Biorad) to remove residual Ca^2+^. CaCl_2_ (1M solution, Fluka) was diluted in size exclusion buffer (10 mM HEPES, 150 mM NaCl, 0.05% Tween, pH 7.4) and a series of 16 dilutions (1:2) was prepared and mixed with protein, resulting in ligand concentrations ranging from 0.6 μM to 200 mM. Proteins were used in concentrations of 1–4 μM. For the negative control 50 or 500 mM EDTA was added to the reaction mixture. The samples were filled into NT.LabelFree Standard Treated Capillaries (NanoTemper Technologies GmbH). The measurement took place at 22°C with laser off/on/off times of 5, 39, or 5 s. The instrument parameters were adjusted to 5% LED power and 20% MST power. The data presented here are from three technical replicates done in the same day; the whole experiment was repeated at least three times confirming the results. We analyzed the temperature jump (fluorescence change during the first second of IR radiation exposure) for each sample.

Fluorescence change during temperature jump was plotted against ligand concentration and curves were fitted with the Hill Fit:

f(c) = unbound + (bound - unbound)/(1 + (EC50/c)^^^n)

in *IGOR* (Wavemetrics).

## Results

### A Pull-Down from Chicken Utricle Reveals CaMKIIδ as a Novel Otoferlin Interaction Partner

In order to identify interaction partners of otoferlin, we used E18 vestibular maculae of the chicken utricle, each containing more than 20000 hair cells for affinity purification of otoferlin. Most vestibular hair cells are functional at this late embryonic age (Goodyear et al., 1999), and utricles can be dissected relatively quickly in larger numbers (Herget et al., 2013). Membrane proteins of 60 avian utricular maculae were solubilized with octyl-β-D-glucopyranoside (Kim et al., 2004) and otoferlin and its potential binding partners were purified using the monoclonal anti-chicken otoferlin antibody HCS-1 (Goodyear et al., 2010), immobilized to magnetic dynabeads. The HCS-1 antibody binds strongly and specifically to chicken otoferlin, but does not recognize mammalian otoferlin.

Specificity of the otoferlin IP was assessed by comparison to a control pull-down using a monoclonal antibody to chicken tip-link antigen protocadherin 15 (Goodyear and Richardson, 2003; Kazmierczak et al., 2007). Eluates of both otoferlin and the control IPs were analyzed by LC-MS/MS. Otoferlin was specifically and efficiently immunoprecipitated with the HSC-1 antibody and 161 otoferlin peptides were identified covering 74% of the chicken utricle protein sequence (Supplementary Figure S1). No otoferlin peptides were found in the control pull-down.

In 11 out of 11 independent IPs, CaMKIIδ co-purified with otoferlin, with at least 4 peptides, 95% peptide threshold, and 99.9% protein probability. CaMKIIδ was not co-purified in the control IPs.

CaMKIIδ is an important modulator of synapses, but has not been described to play a role in the inner ear. We therefore aimed to find out whether CaMKIIδ interacts with otoferlin and phosphorylates it in mammalian auditory hair cells.

**FIGURE 2:**
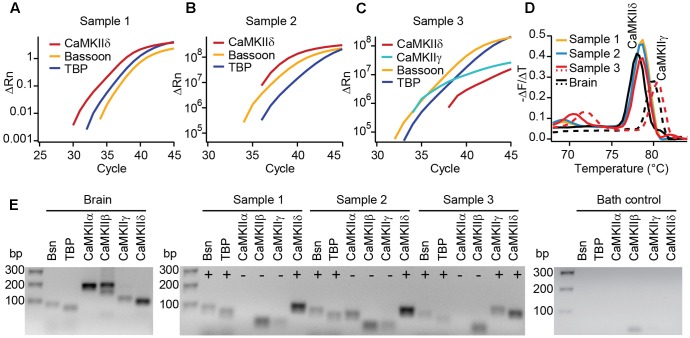
Real-time PCR reveals the expression of CaMKIIδ in mouse IHCs. **(A–C)** Cytoplasm of 3–5 IHCs per sample of P14 mice were collected and analyzed by PCR for the mRNA expression of CaMKIIα, β, γ, and δ. TaqMan assays for bassoon (Bsn) and TATA-binding protein (TBP) were used to control for proper cDNA quality. In three out of three samples, SYBR green fluorescence indicates the expression of CaMKIIδ mRNA; in one sample **(C)** CaMKIIγ mRNA was expressed in addition. We did not find CAMKIIα or β transcripts in any of the samples. **(D)** Melting curve analysis (derivative of melting curve is displayed) for the SYBR green assays of the three IHC cDNA samples and brain cDNA samples for comparison; the amplicons using brain cDNA as template revealed the proper melting temperature of the CaMKIIδ and γ amplicons from IHC samples. **(E)** Amplicons from positive control experiments on brain cDNA, amplicons from experiments in **(A–C)** and one representative bath control were analyzed by electrophoresis on 2% agarose gels with EtBr staining. Correct sized amplicons were found for bassoon and TBP in the brain and in all three IHC samples, but not in the bath control. Primers for CaMKIIα and CaMKIIβ did not give PCR products of correct size in samples 1–3. CaMKIIγ could be amplified from sample 3 only. The transcripts for CaMKIIδ were present in samples 1, 2, and 3, displayed by amplicons of the correct size.

We investigated the localization and expression of all CaMKIIs in mammalian IHCs using immunohistochemistry (**Figure 1**) and PCR (**Figure 2**).

In mouse organs of Corti at P14 we found hardly any immunolabelling for CaMKIIα, CaMKIIβ or CaMKIIγ within the cytoplasm of IHCs, which were co-labeled for otoferlin (**Figures 1A–C**). Immunolabelling against CaMKIIβ, CaMKIIγ and CaMKIIδ appeared outside of IHCs, possibly in efferent and/or afferent synaptic boutons (**Figures 1B–D**). For CaMKIIδ, immunoreactivity could be detected also within the cytoplasm of IHCs (**Figure 1D**). To narrow down the localization of CaMKIIδ at the synapse, we co-labeled with the ribbon protein Ctbp2 and the postsynaptic marker PSD95. Immunolabelling revealed CaMKIIδ to localize close to PSD95, at the opposite side of the ribbon (**Figure 1E**), indicating a postsynaptic localization of CaMKIIδ in afferent synaptic boutons, in addition to the cytoplasmic localization within the IHCs.

To test for different CaMKII mRNA transcripts in IHCs, we designed PCR primers specific for each of the four CaMKII genes. Suitability to amplify the respective CaMKII transcripts was confirmed using mouse brain cDNA as template in real-time PCR with SYBR green (**Figure 2E**). 3–5 IHCs per sample were collected with a patch pipette and mRNA was reverse transcribed. Only samples displaying TaqMan-PCR signals for bassoon and TATA-binding protein as housekeeping genes were considered for analysis. Real-time PCR experiments revealed the presence of CaMKIIδ transcripts in three independent samples (**Figures 2A–C**). CaMKIIγ mRNA could be detected in one out of three samples (**Figure 2C**), while transcripts from CaMKIIα and CaMKIIβ could not be amplified in any IHC sample (**Figure 2E**). Therefore, we conclude that CaMKIIδ is the predominant CaMKII in rodent IHCs, with a supporting contribution by CaMKIIγ.

### A Proximity Ligation Assay Confirms Molecular Interaction of Otoferlin and CaMKIIδ in Rat Cochlear IHCs

**FIGURE 3:**
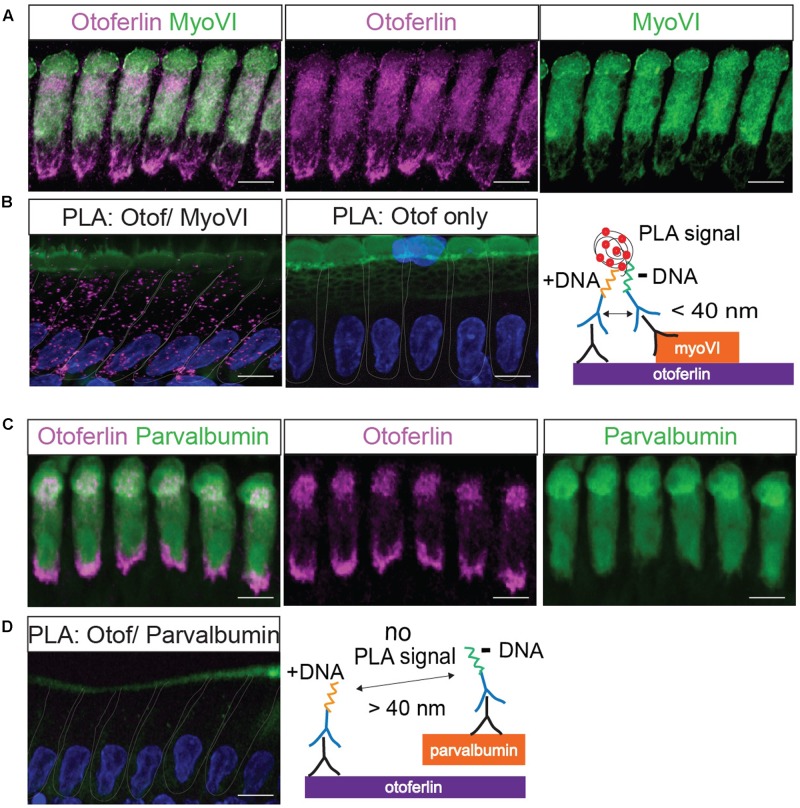
Validation of a proximity ligation assay (PLA) by visualization of the protein interaction of otoferlin and myosin VI *in situ*. **(A)** Z-projections of confocal sections of IHCs from a whole-mount explant of a P11 rat organ of Corti, immunolabeled for otoferlin and myosin VI. **(B)** Primary antibodies from **(A)** were used for a PLA (see cartoon) to detect *in situ* protein interactions (magenta punctae in left panel) of otoferlin with myosin VI within 40 nm distance. No PLA puncta were detected when only one primary antibody to otoferlin was used for PLA (middle panel). **(C,D)** Immunohistochemistry and negative control for the PLA protocol. **(C)** Z-projections of confocal sections of IHCs from a P11 rat organ of Corti whole-mount explant, immunolabeled for otoferlin and parvalbumin. **(D)** Primary antibodies from **(C)** were used for a PLA. No PLA puncta were detectable suggesting a lack of otoferlin-parvalbumin interaction. In **(B,D)** cell nuclei were stained with DAPI (blue), hair cell stereocilia with Phalloidin (green). Scale bar, 10 μm.

We next investigated whether CaMKIIδ and otoferlin interact in mammalian IHCs using an immunohistochemistry based *in situ* proximity ligation assay (PLA) (**Figure 3**) which detects a <40 nm proximity of antibody-labeled proteins (Koos et al., 2014).

First, we validated the PLA with a previously reported interaction of otoferlin with myosin VI (**Figure 3**; Heidrych et al., 2009; Roux et al., 2009). We applied the PLA in acutely isolated P11 rat organ of Corti explants resulting in discrete fluorescent puncta distributed over the whole IHC body (**Figure 3B**), indicating close proximity (<40 nm) of otoferlin and myosin VI, likely due to physical interaction. When the PLA assay was done with only one primary antibody to otoferlin as a control (**Figure 3B**, middle panel), no puncta were detected. Similarly, no PLA signals were detectable when we performed the PLA with antibodies to otoferlin and parvalbumin (**Figure 3D** and cartoon), another hair cell marker that – like myosin VI – labels the whole IHC body but is not described as otoferlin interaction partner (**Figure 3C**).

**FIGURE 4:**
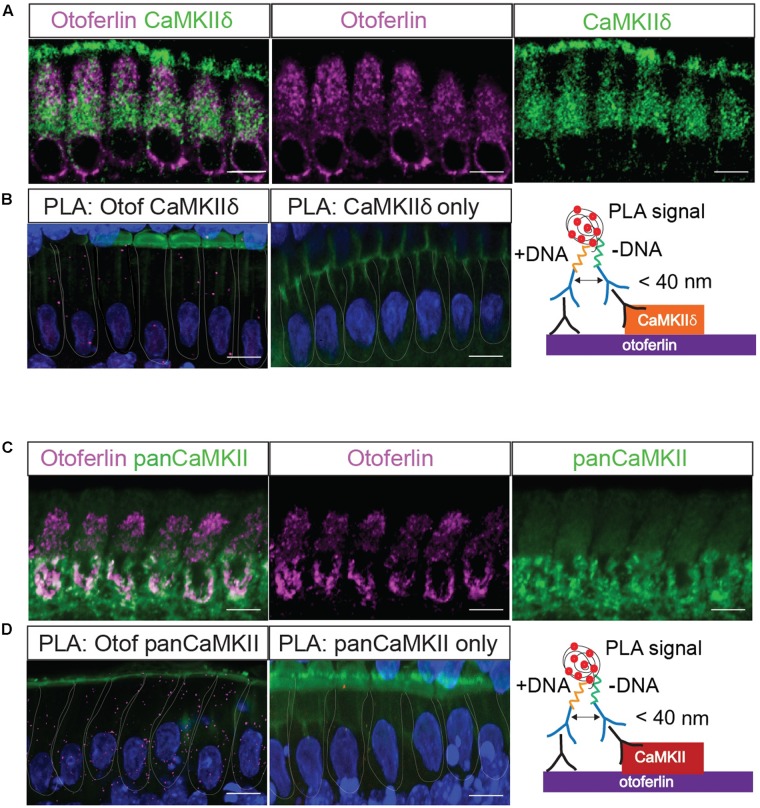
*In situ* interaction of otoferlin and CaMKII demonstrated by PLA. Z-projections of five confocal sections of IHCs from whole-mount explants of P11 rat organ of Corti, immunolabeled for otoferlin and CaMKIIδ **(A)** and otoferlin and pan-CaMKII, respectively **(C)**. The same primary antibodies were used for PLA to detect *in situ* protein interactions of otoferlin with CaMKIIδ **(B)** or all CaMKII isoforms **(D)** using the pan-CaMKII. Magenta punctae display locations of close proximity of the two antibodies. No puncta were detected when only one primary antibody to CaMKIIδ (**B**, middle panel) or pan-CaMKII (**D**, middle panel) were used for PLA as control. Blue: DAPI, green in **(B,D)**: Phalloidin. Scale bar, 10 μm.

Next, we used the PLA to verify a molecular interaction of otoferlin with CaMKIIδ in rat IHCs *in situ* (**Figure 4**). A close proximity of both proteins was indicated by fluorescent puncta in IHCs, suggesting CaMKIIδ to be an otoferlin interaction partner in mammalian cochlear IHCs (**Figure 4B**). PLA puncta also appeared with the pan-CaMKII antibody (**Figure 4D**), which was raised against the kinase domain that is highly conserved between the four CaMKII genes. No PLA signals were detected in control assays, using anti-CaMKII antibodies only (**Figures 4B,D**, middle panels).

### CaMKIIδ Binds Otoferlin *In Vitro*

**FIGURE 5:**
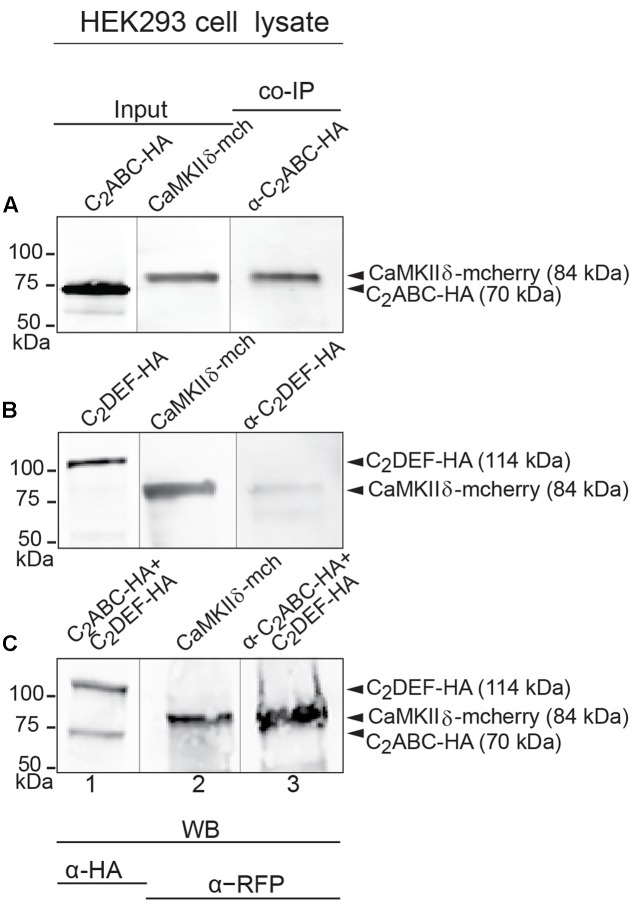
Immunoprecipitation and western blot show interaction of otoferlin with CaMKIIδ. **(A–C)** Two HA-tagged mouse otoferlin fragments, C_2_ABC (aa 1–632 in NP_001093865; 70 kDa) and C_2_DEF (aa 933–1920; 114 kDa) were co-transfected with mcherry-tagged mouse CaMKIIδ into HEK293 cells. Transfections were performed either with otoferlin C_2_ABC and CaMKIIδ (**A**, Input Lane 1 and 2), otoferlin C_2_DEF and CaMKIIδ (**B**, Input Lane 1 and 2) or in the presence of both C_2_ABC and C_2_DEF fragments and CaMKIIδ (**C**, Input Lane 1 and 2). Co-immunoprecipitations of C_2_ABC-HA and C_2_DEF-HA were conducted from HEK293 cell lysates using anti-HA antibodies. CaMKIIδ-mcherry was detected in the eluate using an anti-RFP (red fluorescent protein) antibody (**A–C**, Lane 3), indicating that CaMKIIδ co-precipitated with recombinant otoferlin fragments.

While a positive PLA signal could in principle result from an indirect protein interaction via scaffold proteins, we tested whether CaMKIIδ and otoferlin interact directly *in vitro.* We expressed two HA-tagged fragments of mouse otoferlin, one comprising the first three C_2_ domains (C_2_ABC-HA, 70 kDa), and one comprising the last three C_2_ domains (C_2_DEF-HA, 114 kDa) as well as full-length mcherry-tagged CaMKIIδ (84 kDa) in HEK293 cells and performed IPs with an anti-HA antibody. In western blots, we identified a ∼84 kDa CaMKIIδ-mcherry band in the eluate of the co-IPs indicating a co-purification with otoferlin C_2_ABC (**Figure 5A**). A fainter band was detected in the eluate when CaMKIIδ was co-purified with C_2_DEF (**Figure 5B**), suggesting a weaker interaction. To mimic an interaction with full-length otoferlin, we co-expressed CaMKIIδ-mcherry with both C_2_ABC-HA and C_2_DEF-HA fragments, resulting again in the co-precipitation of the ∼84 kDa band of CaMKIIδ-mcherry in the eluate (**Figure 5C**).

### Otoferlin Is Phosphorylated by CaMKIIδ *In Vitro*

**FIGURE 6:**
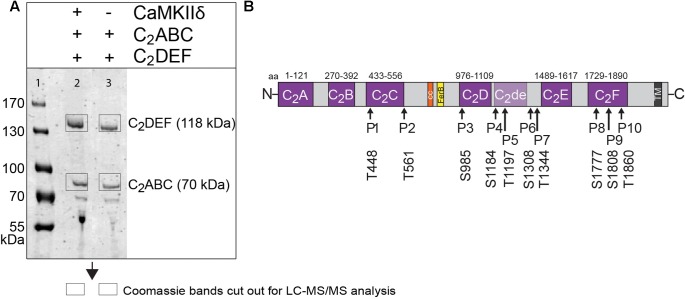
Otoferlin is phosphorylated by CaMKIIδ *in vitro*. **(A)** Otoferlin fragments C_2_ABC (aa 1–616 in NP_001093865, 70 kDa) and C_2_DEF (aa 908–1932, 118 kDa), were expressed in *E. coli* and subjected to an *in vitro* phosphorylation assay with CaMKIIδ and Ca^2+^/calmodulin. Reactions were stopped after 5 min of incubation and proteins were run on a Coomassie gel. Note the slight shift in mass of the fragments between experiment (lane 2) and control without kinase (lane 3). Coomassie stained bands corresponding to otoferlin C_2_DEF and C_2_ABC were cut off the gel and processed for mass spectrometric analysis of otoferlin phosphorylation (Supplementary Figure S2). **(B)** Three independent experiments as in **(A)** revealed 10 serine/threonines in otoferlin that were reproducibly phosphorylated by CaMKIIδ. The putative otoferlin domain topology (in mouse isoform 1; NP_001093865) predicts six C_2_ domains (C_2_A to C_2_F; purple), a coiled-coiled domain (orange), a FerB domain (yellow), and a transmembrane domain (TM) (dark gray). Five of the phosphorylation sites are located in C_2_ domains.

We next performed *in vitro* phosphorylation assays to assess whether CaMKIIδ phosphorylates recombinant otoferlin in a Ca^2+^/calmodulin-dependent manner. We used *E. coli* as an expression system because recombinant proteins produced in bacteria lack phosphorylation (Sahdev et al., 2008). We combined purified otoferlin fragments (C_2_ABC and C_2_DEF) with or without recombinant CaMKIIδ and activated the phosphorylation reaction with Ca^2+^ and calmodulin. After incubation for 5 min, otoferlin C_2_ABC and C_2_DEF fragments were analyzed for phosphorylation by LC-MS/MS after in-gel trypsinization (**Figure 6A**). Phosphorylation sites were identified by 80 Da mass shifts in the respective peptides (Supplementary Figure S2).

**FIGURE 7:**
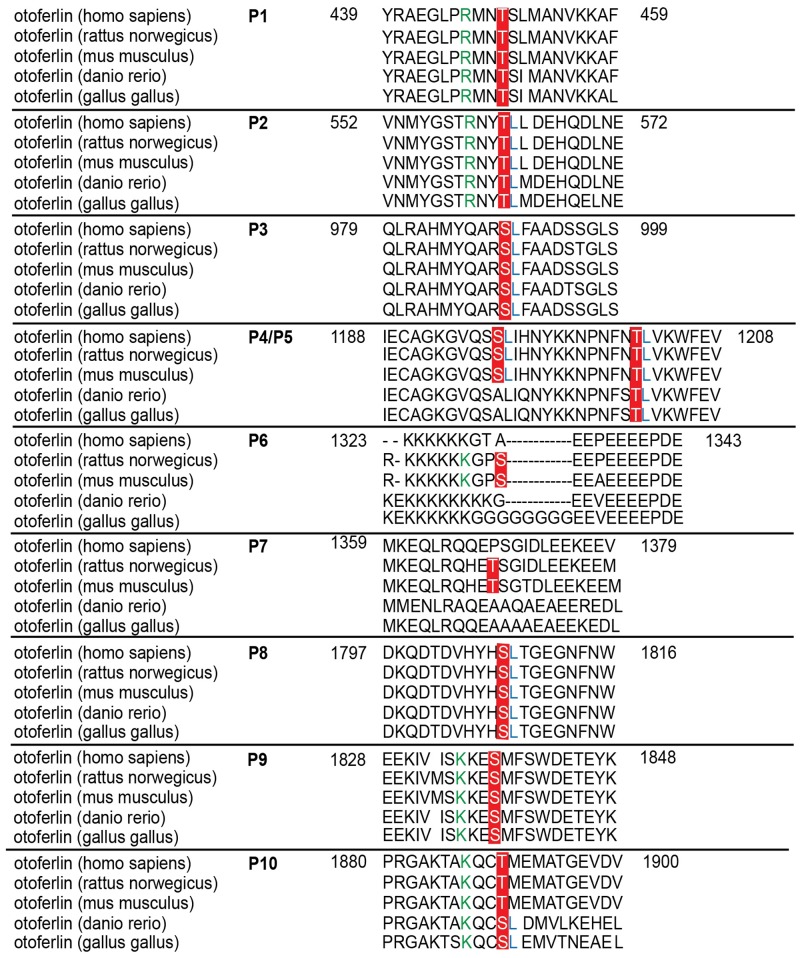
Sequence alignment of phosphorylated sites for otoferlin from different species. Sequence alignment of phosphorylated mouse otoferlin peptides (NP_001093865) with human (NP_919224), rat (XP_006239895), zebrafish (NP_001025283), and chicken (XP_420015) otoferlin. As indicated in red, seven of the phosphorylation sites are conserved amongst the species (P1, P2, P3, P5, P8, P9, and P10). P6 and P7 are only found in rat and mouse otoferlin and P4 is conserved amongst human, rat and mouse. Potential CaMKII consensus motifs (R/K-X-X-S/T) are indicated by green arginines (R) or lysines (K) at the -3 position of the phosphorylation sites. Hydrophobic leucine residues at the +1 position of a phosphoserine or -threonine that were shown to be favored by CaMKII (White et al., 1998) are labeled in blue.

**FIGURE 8:**
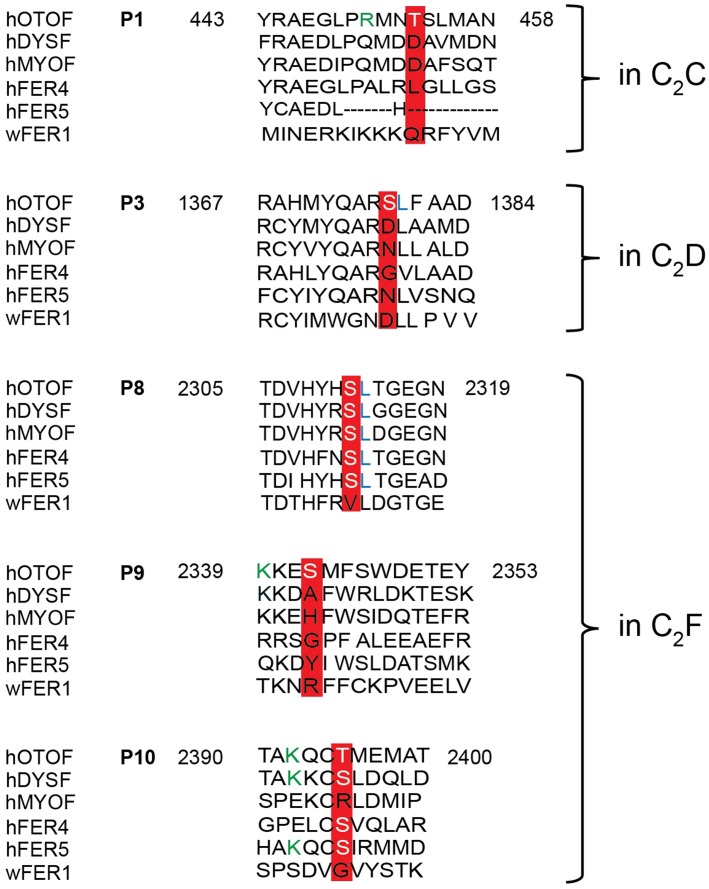
Sequence alignment of otoferlin C_2_ domain phosphopeptides with other ferlin proteins. Phosphoserines and -threonines that were identified within an otoferlin C_2_ domain were aligned with human dysferlin (hDYSF, O75923), myoferlin (hMYOF, Q9NZM1), Fer4 (ALFER1L4), Fer5 (LOC90342) and worm Fer1 (Q17388), according to Jiménez and Bashir (2007). Conserved phosphosites are only found in the C_2_F domain of human otoferlin paralogs, including the phosphoserine of P8 and the phosphothreonine of P10. Lysines (K) of a potential CaMKII consensus motif (R/K-X-X-S/T) in P9 and P10 are indicated in green font color.

Only phosphorylation sites identified in three independent experiments were considered. We found that both the N-terminal otoferlin C_2_ABC fragment as well as the C-terminal C_2_DEF fragment were phosphorylated when incubated with CaMKIIδ *in vitro* and lacked phosphorylation in the absence of the kinase. In total, we identified ten phosphorylation sites (P1 to P10, **Figure 6B**). Five sites are located within C_2_ domains of otoferlin, including T448 (C_2_C domain), S985 (C_2_D domain), and S1777, S1808, T1860 (C_2_F domain; C_2_ domain borders according to Jiménez and Bashir, 2007). S1184 and T1197 were identified in a region between the C_2_D and the C_2_E domains which has been hypothesized to fold as a C_2_ domain (C_2_de; Washington and Ward, 2006; Han and Campbell, 2007; Pangršič et al., 2012). Most of the identified phosphorylation sites were found to be conserved between mammalian and non-mammalian otoferlin orthologs (**Figure 7**), but only a few are conserved within C_2_ domains of the ferlin protein family (**Figure 8**). Five out of the ten phosphorylation sites followed the CaMKII consensus sequence R/K-X-X-S/T (White et al., 1998). Noticeably, in six phosphopeptides a hydrophobic leucine was found at the P+1 site of the phosphoserine or threonine (indicated in blue in **Figures 7**, **8**), displaying a preferred residue for CaMKII recognition (Stokoe et al., 1993).

### Phosphorylation by CaMKIIδ Alters the Ca^2+^ Affinity of Recombinant Otoferlin C_2_C and C_2_F Domains

**FIGURE 9:**
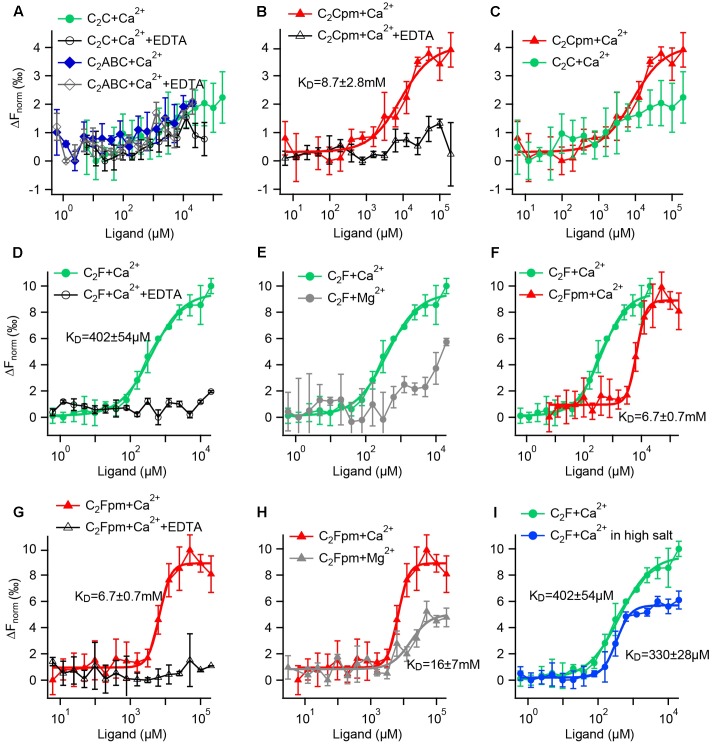
Microscale thermophoresis (MST) assays reveal that phosphorylation increases the Ca^2+^ affinity of the C_2_C domain and reduces the Ca^2+^ affinity of the C_2_F domain. **(A–I)** Fluorescence changes after infrared laser mediated heating of the sample indicate binding of a ligand. Data points are mean values ± SD for *n* = 3 technical replicates each. K_D_s were acquired by Hill fitting (solid lines) in *IGOR* (Wavemetrics). **(A)** A minor change in fluorescence for the C_2_C domain or a fragment containing the C_2_ABC domains did not differ from the negative controls with EDTA, suggesting no Ca^2+^ binding. **(B)** In contrast, the phosphomimetic (pm) C_2_C domain (T449D) showed binding to Ca^2+^, but with rather low affinity. **(C)** Direct comparison for the wild-type and the phosphomimetic C_2_C domain illustrates that phosphorylation increases the Ca^2+^ affinity. **(D)** The wild-type C_2_F domain binds to Ca^2+^; but not when EDTA was present. **(E)** In comparison, the binding of Mg^2+^ to the C_2_F domain is much weaker than the binding to Ca^2+^. **(F)** Compared to the non-phosphorylated C_2_F domain, the three phosphomimetic mutations lower Ca^2+^ affinity by one order of magnitude; **(G)** no binding occurred in the presence of EDTA. **(H)** Mg^2+^ affinity is lower than Ca^2+^ affinity for the phosphomimetic C_2_F domain. **(I)** Measuring in a high salt buffer (300 mM NaCl) slightly lowers the K_D_ for Ca^2+^.

According to the comparison between ferlin protein family members, the position of the phosphorylated threonine at P1 is occupied by negatively charged aspartate residues in dysferlin and myoferlin (**Figure 8**), which are positioned in the top loops of the C_2_ domain, just next to aspartate residues predicted to coordinate Ca^2+^ (Jiménez and Bashir, 2007). We therefore addressed the influence of CaMKIIδ phosphorylation on the Ca^2+^ affinity of the recombinant otoferlin C_2_C domain. To mimic the negative charge introduced by phosphorylation, we mutated threonine residue T448 into an aspartate residue (T448D). Ca^2+^ affinity was assessed by microscale thermophoresis (van den Bogaart et al., 2012) (**Figure 9**).

For the non-phosphorylated C_2_C domain no changes in fluorescence were detected for Ca^2+^ concentrations between 6 μM and 200 mM compared to the negative control carried out in the presence of 500 mM EDTA (**Figure 9A**). Thus, either Ca^2+^ binding occurred but did not change the thermophoresis signal, or the non-phosphorylated C_2_C domain did not bind to Ca^2+^ in this assay. Accordingly, the recombinant otoferlin C_2_ABC construct revealed no change in thermophoresis signal, suggesting lack of Ca^2+^ binding (**Figure 9A**). For the phosphomimetic C_2_C domain (T448D) we observed a fluorescence change above the one triggered by Ca^2+^ plus EDTA (**Figure 9B**). Curve fitting resulted in an apparent dissociation constant (K_Ca_) of 8.7 ± 2.8 mM. Hence, phosphorylation by CaMKIIδ most likely converts the C_2_C domain from a non-Ca^2+^ binding into a Ca^2+^ binding C_2_ domain (**Figure 9C**), although with rather low Ca^2+^ affinity.

Next, we assessed the effect of phosphorylation on Ca^2+^ affinity of the C_2_F domain. For the non-phosphorylated C_2_F domain, we found an apparent dissociation constant for Ca^2+^ of 402 ± 54 μM, which was abolished in the presence of EDTA (**Figure 9D**). Using Mg^2+^ instead of Ca^2+^, we also detected a change in fluorescence, yet a Mg^2+^ concentration of 20 mM was not sufficient to reach a plateau, indicating a rather low Mg^2+^ affinity of the C_2_F domain (**Figure 9E**). We then mimicked phosphorylation of P8, P9 and P10 by replacing the respective serine/threonine residues by aspartates (S1777D, S1808D, T1860D). The phosphomimetic C_2_F domain binds Ca^2+^ with a K_Ca_ of 6.7 ± 0.7 mM (**Figure 9F**, corresponding negative control: **Figure 9G**). For Mg^2+^ we obtained an apparent dissociation constant of 16.2 ± 7.2 mM (**Figure 9H**), which is at least one order of magnitude above the intracellular concentration of free Mg^2+^ (<1 mM) (Romani and Scarpa, 1992). To assess the effect of salt concentration on the C_2_F domain stability and hence Ca^2+^ affinity, we measured the K_Ca_ with 150 mM NaCl as above (**Figure 9D**) compared to 300 mM NaCl. As indicated in **Figure 9I**, the fluorescence changes reached a plateau earlier for high salt concentrations, resulting in a lower apparent K_Ca(high salt)_ of 330 ± 28 μM.

In summary, phosphomimetic mutations decreased the Ca^2+^ affinity of the C_2_F domain by at least one order of magnitude. This suggests that phosphorylation by CaMKIIδ likely results in a lower Ca^2+^ affinity of the otoferlin C_2_F domain.

### A PLA Reveals Activity-Dependent Phosphorylation of Otoferlin Protein Complexes by CaMKII in IHCs

**FIGURE 10:**
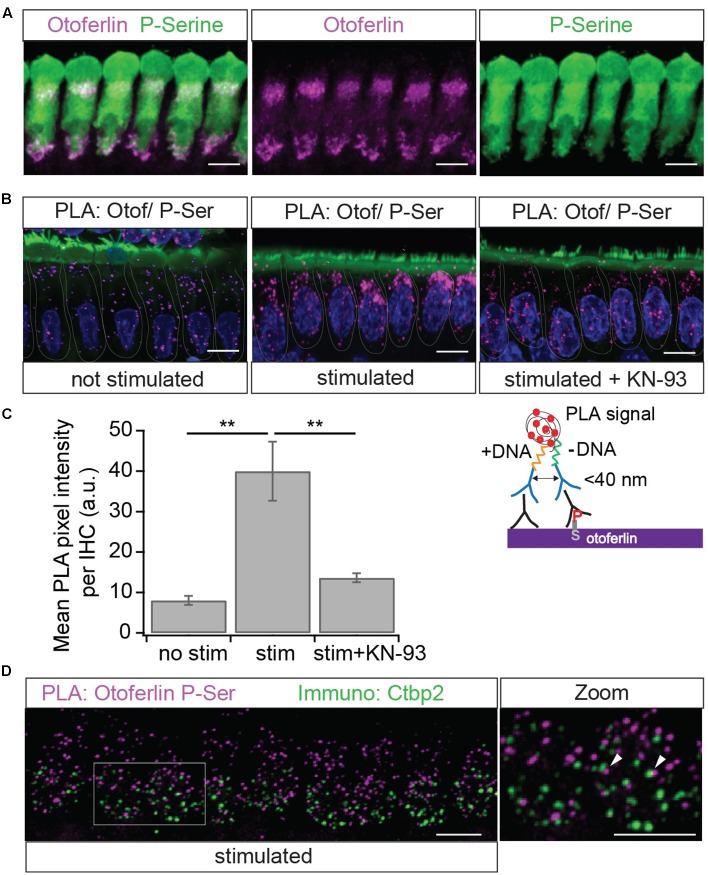
Immunolocalization of phosphoserine residues close to otoferlin in IHCs. **(A)** Projection of a confocal z-stack of IHCs from a whole-mount explant of a rat P10 OC immunolabeled for otoferlin (magenta) and phosphoserine (green). **(B)** Primary antibodies from **(A)** were used to detect phosphoserine residues in <40 nm distance from otoferlin *in situ* by PLA. Nuclei staining in blue (DAPI) and hair cell stereocilia in green (Phalloidin). Magenta PLA punctae indicate phosphoserine residues on otoferlin and/or on a direct interaction partner after 15 min at 37°C in low extracellular KCl solution (left panel). Hair cell stimulation with 40 mM KCl for 15 min at 37°C (middle panel) appears to increase PLA signals compared to not stimulated hair cells. Incubation with the CaMKII inhibitor KN-93 (right panel) blocks this effect. **(C)** Quantification of the PLA signal intensities. Compared with resting conditions, hair cell stimulation significantly increases PLA fluorescence intensity (two-tailed *t*-test, *p* = 0.0017; three independent experiments, with 30 IHCs analyzed for each condition) and pharmacological inhibition of CaMKII with KN-93 blocks most of this effect (two-tailed *t*-test, *p* = 0.0034; *N* = 3 experiments with 30 IHCs for each condition). Bars show mean ± SD; two-tailed *t*-test; ^∗∗^*p* = 0.001 – 0.01. **(D)** Projection of a confocal z-stack of IHCs from a whole-mount explant of rat P9 organs of Corti after PLA assay with primary antibodies to otoferlin and phosphoserine (magenta) and a parallel immunostaining against the ribbon marker Ctbp2 (green) indicates close proximity but no co-localization of phosphorylated otoferlin protein complexes and synaptic ribbons. Scale bars: 10 μm.

Using a PLA to find phosphoserine residues in <40 nm distance from otoferlin, we next tested whether otoferlin and/or proteins interacting with otoferlin are phosphorylated in IHCs (**Figure 10**). To assess whether otoferlin phosphorylation depends on hair cell activity *in vivo*, we applied low or high external K^+^ solutions to acutely isolated OCs. High K^+^ depolarizes the plasma membrane leading to opening of voltage gated Ca^2+^ channels, and the Ca^2+^-influx triggers Ca^2+^-induced exocytosis but is also predicted to activate CaMKII by the Ca^2+^/calmodulin complex. We found PLA punctae in IHCs at resting conditions (**Figure 10B**), indicating a basal level of phosphorylated otoferlin or otoferlin interaction partners. After exposure to high K^+^, we observed more and brighter PLA signals (**Figure 10B**, middle panel). Comparing mean pixel intensities of the fluorescent PLA signals in resting IHCs with the ones in IHCs after stimulation, we found a four-fold fluorescence increase in high K^+^-stimulated IHCs (**Figure 10C**). This suggests a higher degree of otoferlin phosphorylation per otoferlin molecule, a higher number of phosphorylated otoferlin molecules, a higher degree of phosphorylation of proteins interacting with otoferlin, an increased interaction of otoferlin with a phosphorylated protein, or a combination of these possibilities.

To assess whether this increase in phosphorylation of otoferlin or the otoferlin interactome is mediated by CaMKII in rat IHCs, we pre-incubated acutely dissected OCs with the cell-permeable CaMKII inhibitor KN-93, which competitively interacts with the Ca^2+^/calmodulin-binding site on CaMKII, (Sumi et al., 1991). In the presence of KN-93, the stimulation-dependent increase in PLA signal was blocked to a large extent (**Figures 10B,C**), suggesting that activity-dependent phosphorylation of otoferlin or the otoferlin interactome in rat IHCs is indeed mostly due to the CaMKII kinase reaction.

Because of its presumed function in synaptic transmission, we next analyzed if phosphorylated otoferlin or the phosphorylated protein complex localizes to synaptic ribbons. We performed the PLA assay as before with 15 min high K^+^-stimulation and co-stained against the ribbon marker Ctbp2 (**Figure 10D**). PLA puncta, likely reflecting phosphorylated otoferlin and/or otoferlin bound to a phosphorylated protein, did not co-localize with the synaptic ribbons, but were rather found to be in the vicinity or adjacent to each other. This suggests that CaMKIIδ regulates otoferlin activity near the active zone membrane or in endocytotic compartments which are both in close proximity to the synaptic ribbon (Duncker et al., 2013; Neef et al., 2014; Revelo et al., 2014; Jung et al., 2015).

In summary, protein complexes containing otoferlin seem to be phosphorylated adjacent to synaptic ribbons *in vivo*. Phosphorylation is strongly promoted by hair cell stimulation and can be blocked by a CaMKII inhibitor.

## Discussion

Otoferlin has been implicated in IHC synaptic vesicle fusion, fast synaptic vesicle replenishment potentially including priming and active zone clearance, in vesicle reformation and clathrin-mediated endocytosis (Roux et al., 2006; Pangršič et al., 2010; Duncker et al., 2013; Jung et al., 2015; Strenzke et al., 2016), suggesting a multifunctional role in synaptic transmission at the hair cell afferent fiber synapse. Here, we provide evidence that CaMKIIδ phosphorylates otoferlin via direct protein interaction. As phosphorylation altered the Ca^2+^ affinity of recombinant otoferlin C_2_ domains, we conclude that CaMKIIδ likely regulates its function.

In a co-purification assay from chicken utricles, we identified CaMKIIδ, a Ca^2+^/calmodulin-dependent serine/threonine kinase, as a binding partner of otoferlin. A direct interaction of mammalian otoferlin and CaMKIIδ was supported by pull-downs of recombinant otoferlin and CaMKIIδ from HEK293 cells and an immunohistochemistry based PLA on acute explants of OCs demonstrating a close proximity of both proteins (<40 nm) in IHCs. CaMKII accounts for 1–2% of all proteins in the brain and is a key modulator of synaptic transmission, mainly through its postsynaptic action (Lisman et al., 2002; Liu and Murray, 2012; Herring and Nicoll, 2016). At the presynaptic site, CaMKII phosphorylates a variety of proteins, including the synaptic vesicle proteins synapsin I, syntaxin 1A, synaptotagmin I as well as Ca_v_1 L-type calcium channels, thereby modulating synaptic vesicle trafficking and exocytosis (Llinás et al., 1991; Fukunaga et al., 1995; Hilfiker et al., 1999; Ohyama et al., 2002; Abiria and Colbran, 2010; Jenkins et al., 2010). However, a recent proteomics study on synaptosomes uncovered no phosphorylation site to be induced by depolarization and Ca^2+^ entry within a C_2_ domain (Kohansal-Nodehi et al., 2016). Concordantly, reports about biochemical regulations of C_2_ domains are rare, e.g., the non-Ca^2+^ binding C_2_ domain of a novel PKC from Aplysia was reported to display higher phospholipid affinity upon phosphorylation (Pepio and Sossin, 2001). The regulation of Ca^2+^ affinity via C_2_-domain phosphorylation might then be a unique mechanism in the hair cell synapse and/or for the ferlin protein family, where phosphorylation has not been studied to date. Here, we addressed the biochemical effects of phosphorylation on the C_2_C and the C_2_F domains of otoferlin only, but in the future it would be interesting to assess the impact of all ten phosphorylation sites on hearing. Interestingly, CaMKII was shown to be involved in sensory adaptation in different sensory modalities. Activated CaMKII is required for adaptation of touch perception in dorsal root ganglion cells which otherwise turns into pain perception (Yu et al., 2015). In the drosophila olfactory system, phosphorylation of synapsin by CaMKII mediates short-term habituation to odors (Sadanandappa et al., 2013). Notably, CaMKII was found to associate with avian utricular and basilar papilla hair cells as well as with synaptic ribbons in bovine retinal photoreceptors (Uthaiah and Hudspeth, 2010; Kantardzhieva et al., 2011) where it phosphorylates syntaxin 3B (Liu et al., 2014), possibly modulating synaptic transmission at sensory ribbon synapses in vestibular and visual sensation. Since long-term adaptation to constant stimuli is assumed not to play a major role in the auditory system, at least for low or medium sound pressure levels, phosphorylation of otoferlin by CaMKIIδ might lead to upregulation of endocytosis and/or vesicle replenishment, to ensure constant signal transmission. On the other hand, CaMKIIδ-dependent regulation could possibly result in a sensory desensitization in response to very loud sounds by downregulation of exocytosis, potentially a protection mechanism against noise-induced glutamate toxicity. Notably, individual synapses in one cell respond differently in terms of voltage-dependent Ca_v_ channel activation and strength of the Ca^2+^ conductance (Frank et al., 2009; Ohn et al., 2016). It is tempting to speculate that the Ca^2+^-induced phosphorylation regulates exocytosis, vesicle replenishment and retrieval and/or endocytosis differentially at the synapses for high spontaneous or low spontaneous activity neurons. In addition, Ca^2+^-induced Ca^2+^ release (CICR) from intracellular Ca^2+^ stores was reported to reduce sustained vesicle release via a so far unknown mechanism (Castellano-Muñoz et al., 2016). The potential involvement of CaMKIIδ in this pathway could regulate otoferlin activity more distally to the active zone.

Five of the ten phosphorylation sites identified by the *in vitro* CaMKIIδ phosphorylation assay were located within C_2_ domains of otoferlin. In human myoferlin or dysferlin, the position of the phosphorylated threonine in the C_2_C domain is held by an aspartate residue (**Figure 8**), which is predicted to form a Ca^2+^ coordination site (Shao et al., 1998; Ubach et al., 1998; Jiménez and Bashir, 2007). For the non-phosphorylated C_2_C domain and a longer fragment containing C_2_ABC domains, we found no Ca^2+^ binding which is in accordance with *in silico* predictions (Jiménez and Bashir, 2007) but contrasts experimental findings from other groups that applied an autofluorescence assay or isothermal titration calorimetry (Johnson and Chapman, 2010; Padmanarayana et al., 2014).

For the wild type C_2_F domain, we determined an apparent dissociation constant for Ca^2+^ of 330 and 402 μM for high and low salt buffer, respectively, which is in good agreement with a K_D(Ca)_ of 267 μM for the C_2_F domain determined by isothermal titration calorimetry in high salt buffer (Ramakrishnan et al., 2014). However, other groups reported the Ca^2+^ affinity of the C_2_F domain to be ∼25 μM using isothermal titration calorimetry, and ∼20 μM assessing autofluorescence changes (Johnson and Chapman, 2010; Padmanarayana et al., 2014). Note that our C_2_F fragment’s size (aa 1695–1934) is different from the ones used in the aforementioned studies (aa 1720–1885), which would lack one β-strand according to *in silico* predictions (Jiménez and Bashir, 2007). In the <1 μM to <100 μM range, no MST signal was detected, indicating either the absence of such a high affinity Ca^2+^ binding site in our C_2_F fragment or a Ca^2+^ binding event which did not result in a detectable change (as for Synaptotagmin-1 C_2_A, van den Bogaart et al., 2012).

The Ca^2+^ concentrations within Ca^2+^ hotspots at IHC ribbon synapses are estimated to range from >10 μM to >100 μM (Roberts, 1994; Beutner et al., 2001; Wong et al., 2014). Therefore, a K_D_ of a few mM for the Ca^2+^ binding of the C_2_C phosphomimetic mutant, indicates that even the phosphorylated C_2_C domain likely does not bind Ca^2+^ *in vivo*. Also, a dissociation constant of a few hundred μM for the Ca^2+^ binding of the C_2_F domain might seem high, yet similar values were observed *in vitro* for the binding of Ca^2+^ to the recombinant synaptotagmin-1 C_2_B domain (K_D_ ∼200 μM) (Fernandez et al., 2001; Radhakrishnan et al., 2009; van den Bogaart et al., 2012). As the presence of negatively charged phospholipids is known to increase the Ca^2+^ affinity of the C_2_ domains of synaptotagmin and protein kinase C (Brose et al., 1992; Guerrero-Valero et al., 2009; van den Bogaart et al., 2012), we speculate that the affinity of the otoferlin C_2_ domains for Ca^2+^ also increases in the presence of phospholipid membranes. Furthermore, other post-translational modifications or protein–protein interactions might affect the Ca^2+^ affinity. For example, other kinases than those of the CaMKII family might be able to phosphorylate otoferlin. In summary, we hypothesize that Ca^2+^ likely binds to the non-phosphorylated otoferlin C_2_F domain *in vivo*.

Phosphorylation of the C_2_F domain, mimicked here by replacing phosphorylated serine/threonine residues by aspartates, resulted in a more than 10-fold reduction in Ca^2+^ affinity. Even in the presence of phospholipid membranes, we assume that the phosphomimetic C_2_F domain is not capable of binding Ca^2+^ in IHCs.

Although phosphomimetic mutations might differ from actually phosphorylated serine/threonine residues, our data suggest that phosphorylation by CaMKIIδ renders the C_2_F domain grossly Ca^2+^-insensitive in IHCs, providing a molecular mechanism for the suggested regulation of otoferlin activity by CaMKIIδ.

The PLA displaying phosphoserine residues in close proximity (<40 nm) to otoferlin showed an increase signal upon stimulation of the IHCs (**Figure 10**). This experimental setting cannot distinguish between phosphorylated serines within otoferlin and those on proteins interacting with otoferlin. However, since we demonstrate that otoferlin and CaMKIIδ interact and a 5 min co-incubation of both proteins *in vitro* is sufficient to trigger the phosphorylation of otoferlin in ten residues (five of which are serines), we presume that at least part of the PLA signal indicates the phosphorylation of otoferlin itself. Nevertheless, even considering that the assay is detecting phosphoserines on proteins interacting with otoferlin, this points toward a CaMKII-dependent regulation of the otoferlin interactome, probably resulting in the regulation of the IHC synaptic activity.

## Conclusion

Upon hair cell stimulation, Ca^2+^ entering the IHCs activates CaMKIIδ which phosphorylates otoferlin. We hypothesize that this phosphorylation renders the C_2_F domain of otoferlin Ca^2+^ insensitive under physiological conditions, which might regulate the kinetics of exocytosis, vesicle replenishment and/or endocytosis.

## Author Contributions

MH, ER, SH, RF, and AR designed study. MH, SM, AC, FG, CA, and ER performed experiments and analyzed data. MH, ER, SM, and AC wrote manuscript and prepared figures. MH, ER, RF, SH, and AR acquired funding.

## Conflict of Interest Statement

The authors declare that the research was conducted in the absence of any commercial or financial relationships that could be construed as a potential conflict of interest.

## Abbreviations

CaMKIIδ: Ca^2+^/calmodulin-dependent serine/threonine kinase II delta
IHC: inner hair cell
IP: Immunoprecipitation
LC-MS/MS: liquid chromatography tandem mass spectrometry
MST: MicroScale Thermophoresis
OC: organ of Corti
PLA: proximity ligation assay
