# Supplementary material for: Activity-Dependent Phosphorylation by CaMKIIδ Alters the Ca2+ Affinity of the Multi-C2-Domain Protein Otoferlin
Source: Front Synaptic Neurosci. 2017 Oct 4;9:13. doi: 10.3389/fnsyn.2017.00013 (PMC5632675; doi:10.3389/fnsyn.2017.00013)
Supplement: Supplementary file 1 [file Presentation_1.PDF]

Supplementary Material

Activity dependent phosphorylation by CaMKIIδ alters the Ca^{2+}-affinity of the multi-C_2-domain protein otoferlin

Sandra Meese, Andreia P. Cepeda, Felix Gahlen, Christopher M. Adams, Ralf Ficner, Anthony J. Ricci, Stefan Heller, Ellen Reisinger[*] and Meike Herget[6*]

***Correspondence**: Meike Herget: meikeher@gmail.com or Ellen Reisinger: ellen.reisinger@med.uni-goettingen.de

Supplementary Figures

Figure S1

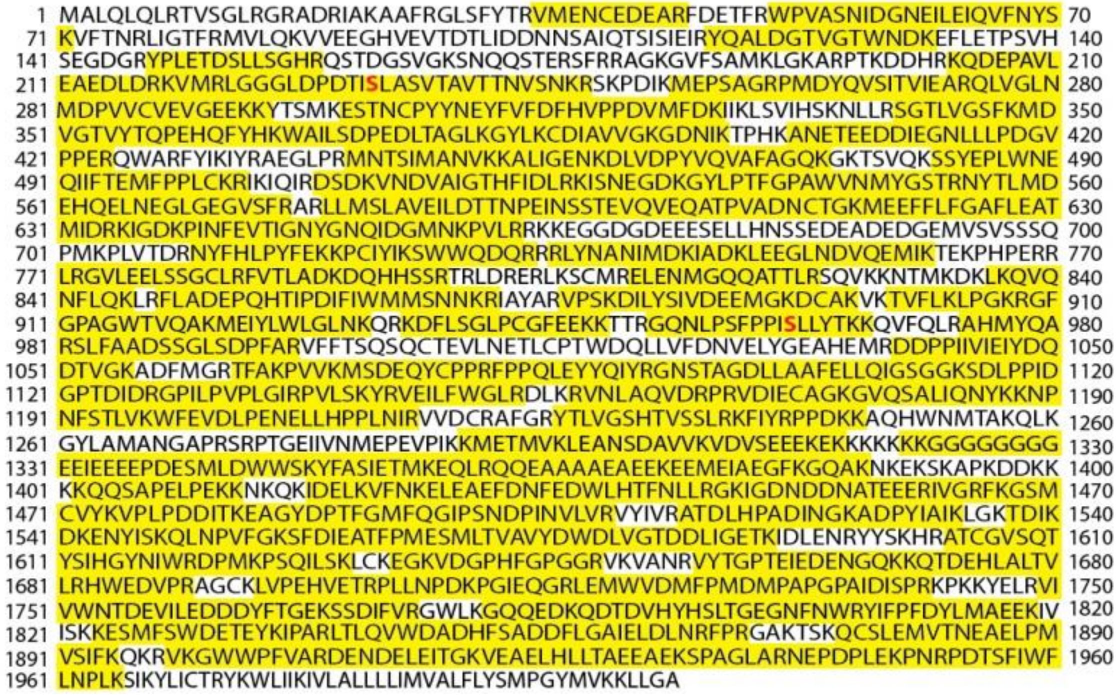

Figure S1. Mass spectrometry identifies 116 peptides of immunoprecipitated otoferlin. Tryptic digestion of immunoprecipitated otoferlin from chicken utricle hair cells generated peptides for mass spectrometric analysis. 116 peptides (yellow) were identified, covering 74% of the chicken otoferlin protein sequence, indicating a specific and efficient protein precipitation by this experimental setup. Two peptides contained phosphorylated serines (shown in red font color).

Figure S2

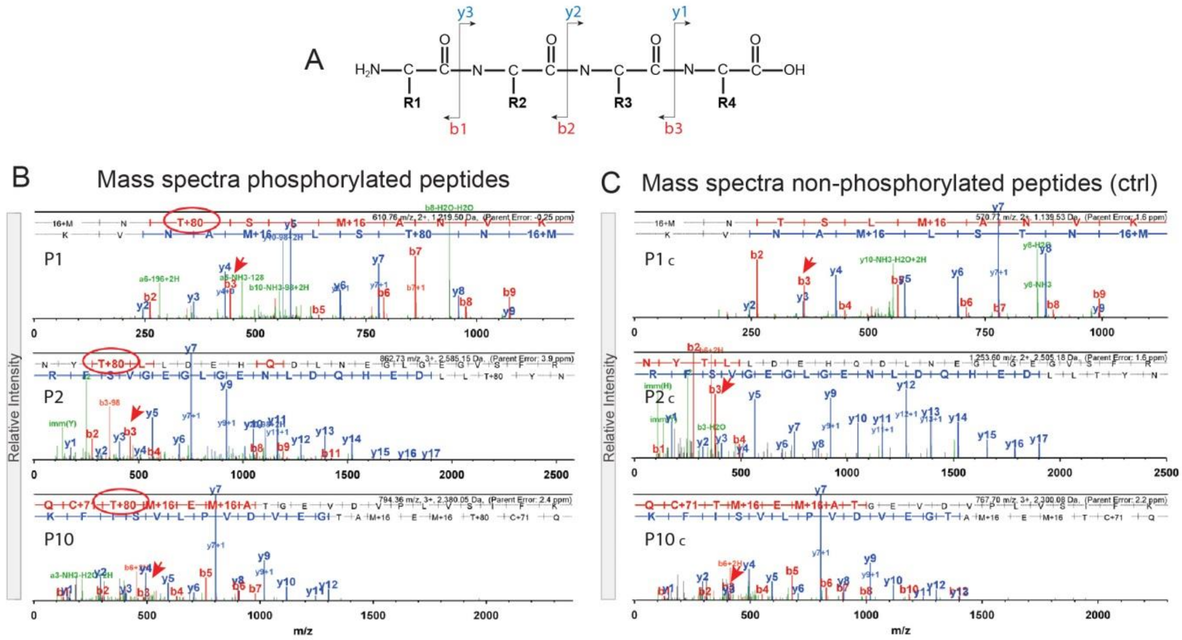

Figure S2: Tandem mass spectrometric analysis of otoferlin peptides after *in vitro* phosphorylation. (A) Nomenclature of common ion types after collision-induced dissociation of peptides by MS/MS. Cleavage at the amide bonds generates b-ions (red) and y-ions (blue), respectively. (B and C) Three representative mass spectra of otoferlin peptides phosphorylated at a threonine (bold **pT**) (P1: MN**pT**SLMANVK, P2: NY**pT**LLDEHQD and P10 QC**pT**MEMATGE) and their corresponding non-phosphorylated control peptides P1c, P2c and P10c, (C). In case of phosphorylation, a mass shift of +80 Da was detected for the given threonine residue, indicated as T+80 (red circle) in the peptide sequence of the P1, P3 and P10 spectra in B. The mass shift of the corresponding b3 ion is indicated by red arrows. Three representative identified phosphorylated otoferlin peptides (P1, P2 and P10) are depicted where collision induced cleavage of the peptide backbone bond generated N-terminal *b*-ions as well as C-terminal *y*-ions as observed in the fragment ion mass spectra (indicated in red and blue, respectively). For the phosphorylation site P1 (MN**pT**SLMANVK), a mass shift of +80 Da was detected for the threonine at the N-terminal position 3 of the peptide sequence, resulting in a shift of the N-terminal *b3*-ion. In the control situation (P1c, without CaMKIIδ, C), no mass shift was detected for the same peptide.
